# Proposal for a new method for sustainable and advanced utilization of oil palm trunk waste

**DOI:** 10.1186/s40643-023-00688-7

**Published:** 2023-10-03

**Authors:** Hiroaki Horiyama, Waka Fujimoto, Keisuke Kojiro, Takafumi Itoh, Hiromu Kajita, Yuzo Furuta

**Affiliations:** Graduate School of Life and Environmental Sciences, Kyoto Prefectural University, Kyoto, 606-8522 Japan

## Abstract

A method to more easily separate vascular bundles and parenchyma was investigated for the purpose of proposing a sustainable and advanced utilization of oil palm trunk (OPT). In addition, particleboard made from vascular bundles was produced as one of the effective ways to utilize the obtained vascular bundles. The following results were obtained. A Zephyr rolling equipment was used for separation, and it was found that the vascular bundles could be easily separated with the veneer in a dry state. SEM observations showed that the vascular bundles could be separated while maintaining the tissue structure. However, some parenchyma remained on the surface of the vascular bundles. The presence of starch was also confirmed within the parenchyma. Particleboard was produced using the separated vascular bundles. The MOR and MOE of the three-layered particleboards with long vascular bundles obtained by Zephyr treatment were about 74.2 MPa and 7.3 GPa, respectively, which are much higher than those of previous wood materials made from OPTs. These results may be the result of extracting the potential of vascular bundles.

**Graphical Abstract:**

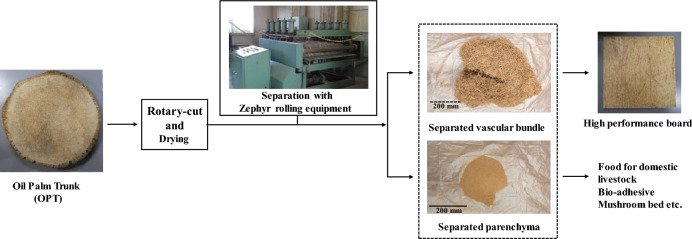

## Introduction

The palm oil industry is thriving in tropical countries, especially in Southeast Asia, where oil palm is grown in vast plantations (Xu et al. 2020). Palm oil is generally used in food industries. The yield of palm oil that can be extracted from oil palm trees decreases after 18 years of age. For this reason, the trees are cut down and replanted after about 25 years (Woittiez et al. 2017). Therefore, an enormous amount of oil palm trunks (OPTs) is generated, and using this waste product as an alternative raw material to produce wood and wood-based products is of great interest (Sulaiman et al. 2012). However, the utilization of felled OPT has not been established, and those are left in the plantations. The green moisture content of the OPT is very high and contains a lot of sugars (Tomimura 1992a; Bahanawan et al. 2019). As a result, the left OPT tends to rot, causing various problems, including problems caused by pests (Abdullah et al. 2012; Pulingam et al. 2022).

The OPT consists mainly of vascular bundle and parenchyma (Tomimura 1992b; Ramle et al. 2012). Vascular bundle is the tissue responsible for the passage function, mainly the vessel and vascular sheath. In the outer zone of cross section, vascular bundles are highly congested but reduced toward the center zone. Along the height, they show an obvious increase from bottom to top (Lim and Khoo 1986). Vascular bundles are stiffer and stronger than parenchyma (Rich 1987; Bahanawan et al. 2019). Parenchyma cells are primarily responsible for the storage function of nutrients, especially starch. (Yusra et al. 2020).

We thought that by separating the vascular bundle and parenchyma, which have different properties, OPT could be used more effectively. For example, vascular bundles could be used as a material such as wood materials, while parenchyma could be used as a source of livestock feed or starch. In a previous report, we have attempted to separate the OPT into vascular bundle and parenchyma and fabricated a particleboard using a separated vascular bundle (Horiyama et al. 2020). The bending strength and Young’s modulus of the particleboard were higher than the particleboard using wood and oil palm chips. It has been suggested that a more accurate separation of vascular bundles and parenchyma from the OPT and control of vascular bundle length may lead to the production of high-performance boards. We focused on the treatment of the zephyr. Zephyr is a sheet material of fibers net-like structure (Kim et al. 1998; Nugroho and Ando 2000). The process involves the progressive crushing of materials through several sets of rollers until a continuous fibrous sheet is obtained (Kikata et al. 1989a, 1989b). The obtained wood and bamboo zephyr strands are used to make strand boards (Kim et al. 1998; Nugroho and Ando 2000; Kikata et al. 1989a, 1989b). There are also reports of zephyr strand boards made from oil palm using the zephyr treatment (Wardani et al. 2014). However, there is no report of using Zephyr treatment for separation.

To propose a new method for sustainable and advanced utilization of oil palm trunks, this study investigated the conditions under which vascular bundles and parenchyma can be more easily separated from OPT by Zephyr treatment. In addition, as one of the ways to utilize the obtained vascular bundles, particleboard made from vascular bundles was produced.

## Materials and methods

### Materials

Rotary-cut OPT veneers of 4 mm in thickness, 460 mm in width, and 1820 mm in length were purchased from a Malaysian plywood manufacturer for plywood production. The size of the veneers for separation of vascular bundles and parenchyma was 4 mm in thickness, 400 mm in width, and 1000 mm in length. The apparent density of oven-dry veneer was approximately 0.31 g/cm^3^. The veneer used in this study was probably taken from the zone except for the outer zone, based on Lim and Khoo’s report (Lim and Khoo 1986). The veneers were dried in a dryer at 80 °C for 2 days. Some of the veneers were used for separation after drying without moisture content adjustment. Other veneers were used for separation after adjusting the moisture content to the air-dry state, fiber saturation point, and water saturated condition by spraying water.

### Separation

**Fig. 1:**
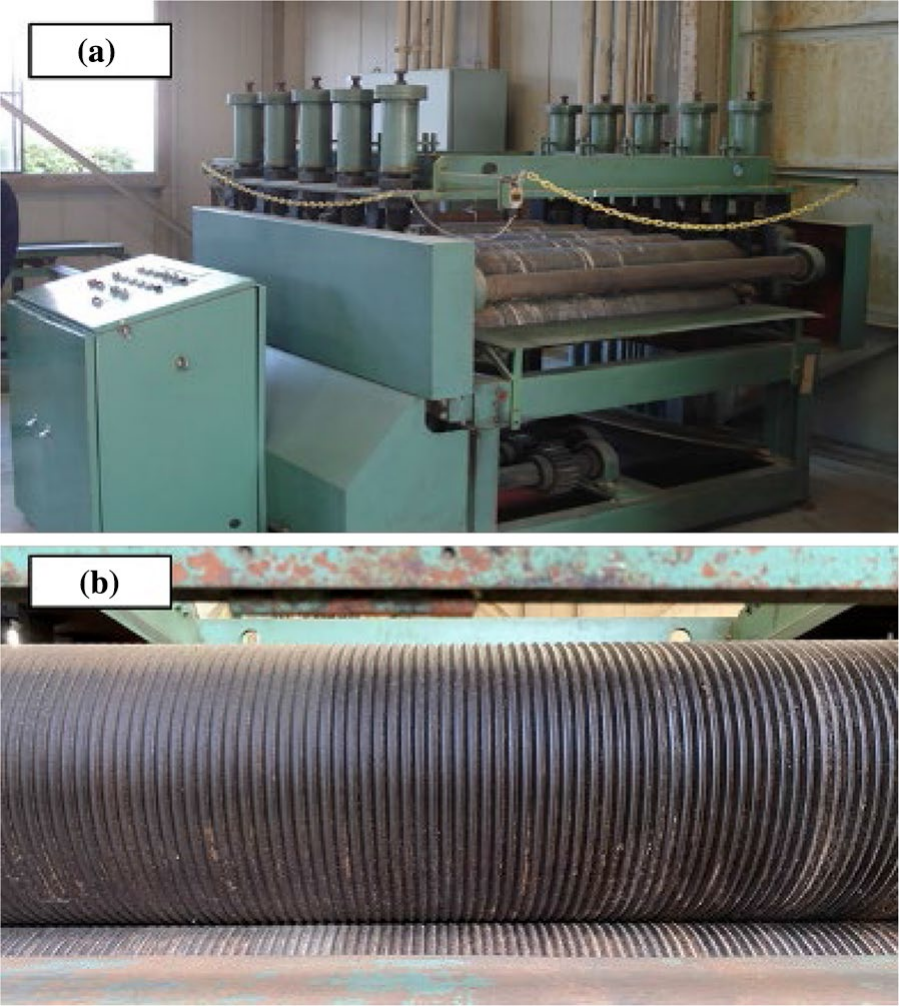
The appearance of Zephyr equipment (**a**) and grooved rollers (**b**)

A Zephyr rolling equipment (Iida Kogyo Co., Ltd) was used at Nara Forest Research Institute in Nara, Japan to separate vascular bundle and parenchyma. Zephyr rolling equipment consists of five pairs of grooved rollers with an outer diameter of 200 mm and a width of 1200 mm, connected to each other as shown in Fig. 1. The upper roller is pressed against the lower roller by the pressure of the cushioning material (hard rubber and spring) on the shaft attached to the upper roller. The roller feed speed was 10 m/min. The separated materials were further sieved to separate fiber and powder. The sieve mesh size was 710 μm.

### Scanning electron microscope (SEM)

The OPT before separation and the separated vascular bundles were observed using SEM (Hitachi High-Technologies, TM3030 Plus Miniscope) at an accelerating voltage of 15 kV. EDX analysis was used energy dispersive X-ray spectrometer (Bruker AXS Co., Ltd, QUANTAX70).

### Fabrication of particleboard

The particleboard produced was 300 mm square and 10 mm thick, and apparent density of 0.7 g/cm^3^. The conditions were random and three-layer orthogonal orientation. The adhesive content was 10 wt.% of oven-dried particles. Acetone was added to as *p*-MDI resin (Aica Kogyo Co., Ltd., AUH-1 4, 4′MDI) by 10 wt.% to decrease its viscosity. The diluted adhesive was sprayed into a revolving blender containing the vascular bundles, which were dried in a dryer at 60 ℃ to about 3%. The manually formed mat was compressed at 180 ℃, 3 MPa for 10 min. A 10 mm distance bar was used to adjust the board thickness. The number of boards produced was one for each condition.

## Mechanical tests

### Bending strength test

Five specimens with dimensions of 280 mm × 50 mm × 10 mm were cut from each particleboard. The three-point bending test was performed using a universal material testing machine (SHIMADZU CORPORATION, AGS-X 10kN) according to JIS A 5908 (2015). The specimens were loaded under a displacement control with a crosshead speed of 10 mm/min and a span of 150 mm. Young’s modulus (MOE) and bending strength (MOR) were obtained using the following formula:

$$\mathrm{MOE}=\frac{{l}^{3}\Delta P}{4b{h}^{3}\Delta y}\left(\mathrm{MPa}\right),$$

$$\mathrm{MOR}=\frac{3Pl}{2b{h}^{2}}\left(\mathrm{MPa}\right),$$

where $P$ and $\Delta P$ are the maximum load and the load at proportional limit. $\Delta y$ is deflection at proportional limit. $b$ and $h$ are the width and thickness of the specimens, respectively. $l$ is the bending span.

### Internal bond (IB) test

Five specimens with dimensions of 50 mm × 50 mm × 10 mm were cut from each particleboard. The test was performed according to JIS A 5908 (2015). Specimens ware glued to attachments with epoxy resign and cured for less than 48 h. The tensile test was performed under a displacement control at a crosshead speed of 2 mm/min. The IB strength was obtained using the following formula:

$$\mathrm{IB}=\frac{P}{bL}\left(\mathrm{MPa}\right),$$

where $b$ and $L$ are the width and length of the specimen, respectively.

### Thickness swelling (TS) test

Five specimens with dimensions of 50 mm × 50 mm × 10 mm were cut from each particleboard. The test was performed according to JIS A 5908 (2015). First, the size and weight of air-dried specimens were measured. Followed by, the specimens were immersed in distilled water at room temperature for 24 h. During this process, the specimens were fixed at a depth of 3 cm under water. Thereafter, the size and weight of the specimens were measured again. The TS was calculated using the following formula:

$$\mathrm{TS}=\frac{{t}_{2}-{t}_{1}}{{t}_{1}}\times 100\left(\%\right),$$

where ${t}_{1}$ and ${t}_{2}$ are thickness of the specimen before and after soaking, respectively.

## Results and discussion

### Separation

**Fig. 2:**
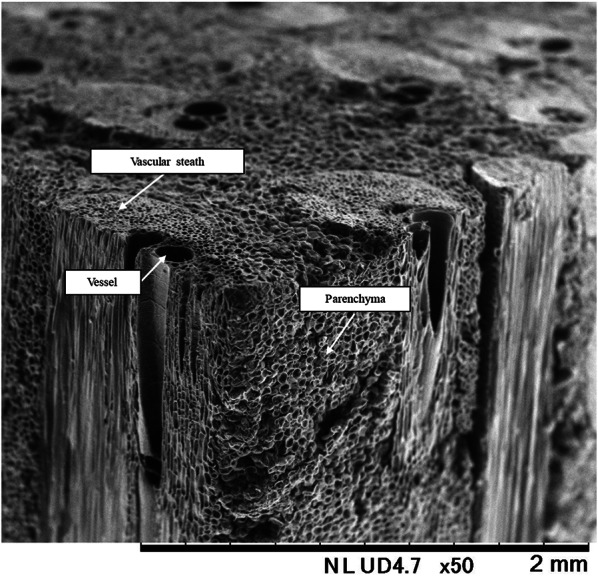
SEM image of longitudinal and transverse section of oil palm trunk

**Fig. 3:**
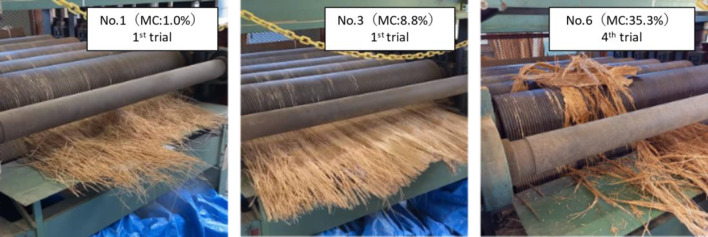
The appearance of the OPT veneer after 1st trial for No.1 and No.3 and 4th trial for No.6 with Zephyr equipment

**Fig. 4:**
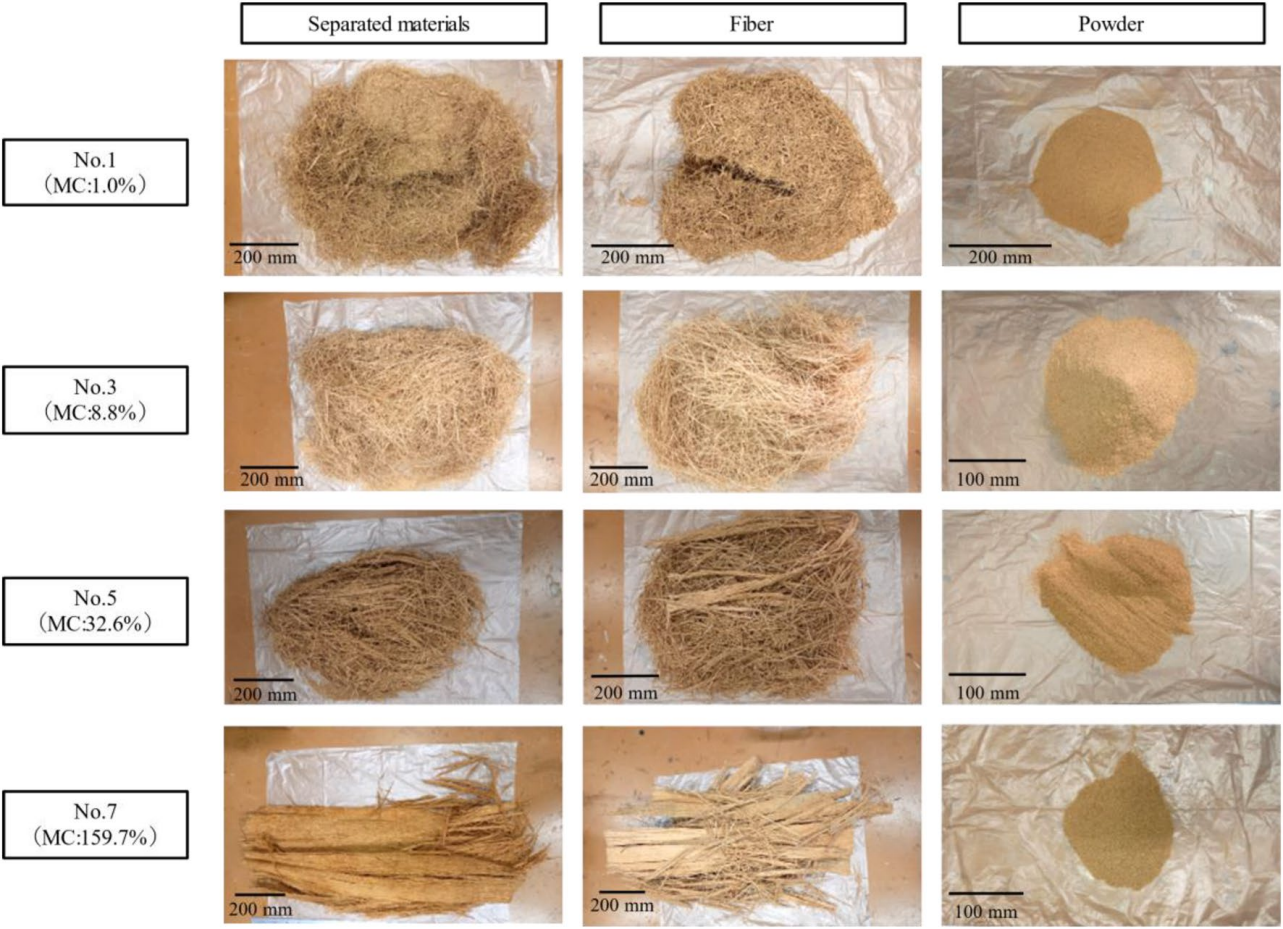
Relationship moisture content of veneer and the appearance of separated materials, fiber, and powder

**Fig. 5:**
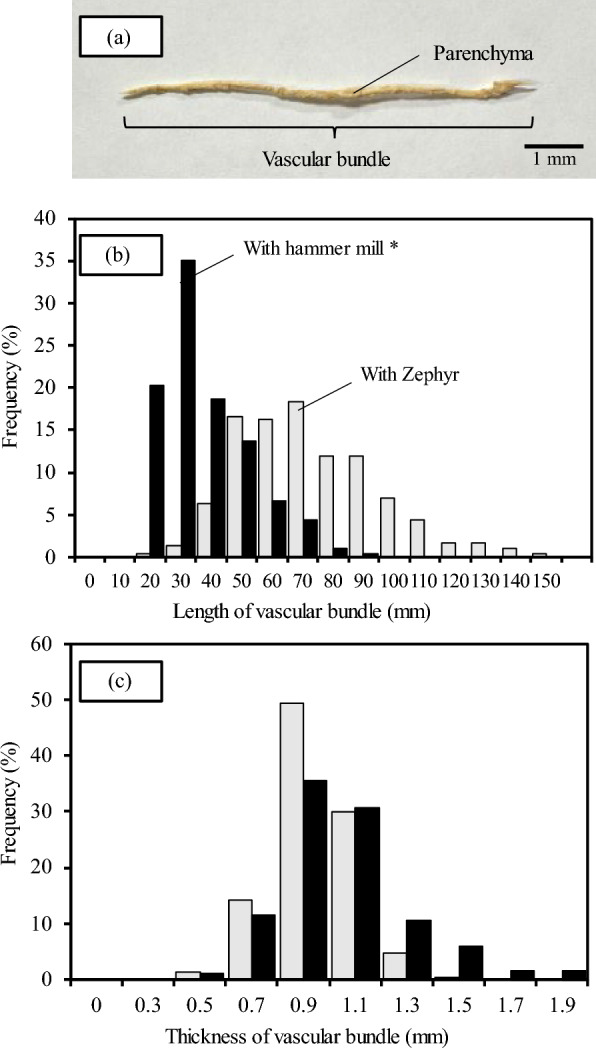
The obtained vascular bundles (**a**) and histograms of the length (**b**) and the thickness (**c**) of vascular bundle. * The data of previous study (Horiyama et al. 2020)

As shown in Fig. 2, the OPT is composed of parenchyma that surrounds vascular bundles. For this reason, the direction of insertion into the Zephyr treatment equipment is thought to affect the ease of separation in the Zephyr treatment of oil palm veneers. Preliminary experiments attempted to separate vascular bundles and parenchyma in directions perpendicular and parallel to the longitudinal direction. However, attempts in the perpendicular direction were unable to separate vascular bundles and parenchyma. Therefore, the below results are given for the parallel direction in which separation was possible. Since the mechanical properties of wood materials are strongly influenced by moisture content, it was considered extremely likely that the moisture content of the veneer would affect the ease of separation. The moisture contents of the veneers used for separation, the yield of the separated materials, and the number of trials required for separation are shown in Table 1. The yield was calculated from the ratio of the weight of separates that could be collected after separation to the weight of the veneer. The veneer with low moisture contents could be easily separated as shown for No.1 in Fig. 3. However, as the moisture contents increased, the separation was difficult. The lower yield of separated materials from the veneer with high moisture contents was due to the water squeezed out during separation and the separates remaining on the rollers as shown for No.6 in Fig. 3. Srivaro et al. reported a gradient in physical and mechanical properties of the OPT within the trunk. According to the observation of the fracture surface of the OPT in the shear test, it was confirmed that parenchyma tore away due to shear force while vascular bundles still stick onto the ground tissue (Srivaro et al. 2018). Therefore, it is reasonable to assume that the grooved rollers of Zephyr equipment were able to separate vascular bundle and parenchyma by causing shear force at their interface. In general, the strength of each wood or woody material decreases with increasing moisture content. This suggested that the reason for the difficulty in separation with increasing moisture contents is due to the decrease in the shear force at the interface between vascular bundles and parenchyma or between parenchymal cells. However, lathe check of veneers and collapse of parenchyma with drying are possible factors in the separation of vascular bundles and parenchyma from the OPT. The appearance of separated materials, fibers, and powder is shown in Fig. 4. Except for No. 7, there were no significant differences in the final separated materials. The appearance of the fibers obtained in No.1 and the histogram of fibers thickness and length are shown in Fig. 5. The fibers obtained by Zephyr treatment were found to have some parenchyma remaining on the surface of the vascular bundles. The length of fibers was mostly between 50 and 90 mm. The thickness of fibers was mostly between 0.9 and 1.2 mm. It was found that the length of the fibers obtained in this study was longer than those of the fibers obtained in the previous report (Horiyama et al. 2020). There are two possible reasons: the length of the veneer used for the separation is long and no breaks have occurred that would shorten the length of the vascular bundle. However, the thickness of the fibers obtained in this study was similar to those of the fibers obtained in the previous report (Horiyama et al. 2020).

**Table 1:** Moisture content of veneer and results of separation

Sample No.	1	2	3	4	5	6	7
MC (%)	1.0	2.0	8.8	8.8	32.6	35.3	159.7
Yield of separated materials (%)	98.1	100	96.4	98.6	95.1	88.4	68.2
Number of trials (count)	1	1	2	3	3	4	-^a^

^a^After the first trial, we found it difficult to separate.

**Fig. 6:**
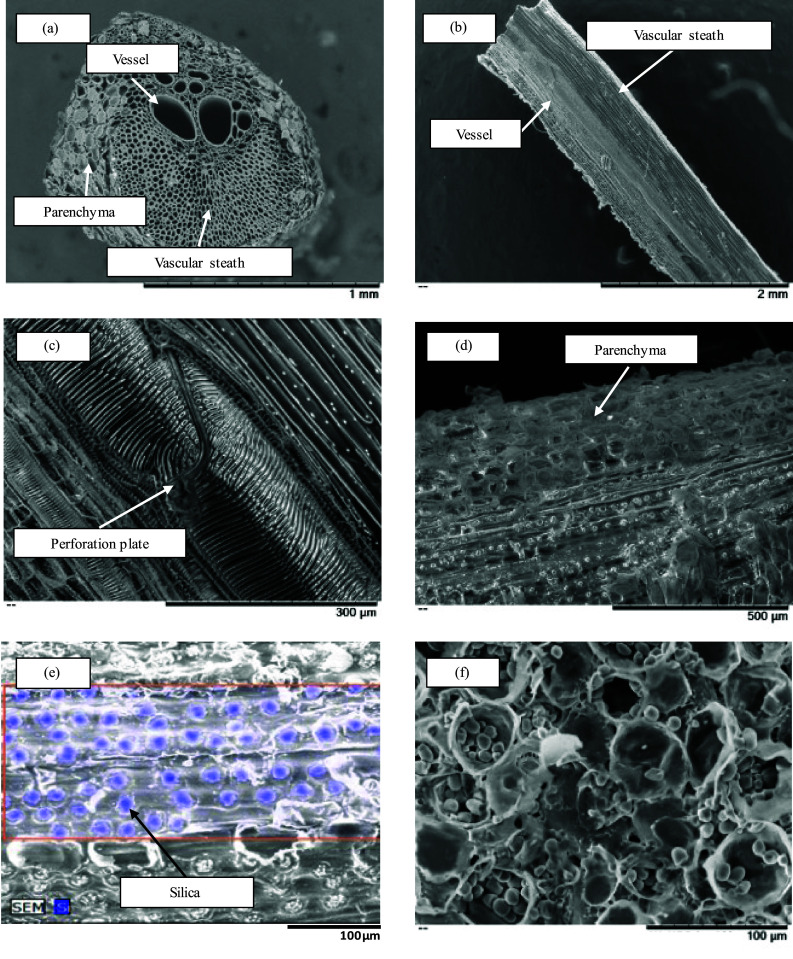
SEM image of vascular bundles separated from oil palm veneer. **a** cross section of vascular bundle, **b** longitudinal section of vascular bundle, **c** near the perforation plate of the vascular bundle, **d** the surface of vascular bundle, **e** EDXA of the surface of vascular bundle, **f** starch in parenchyma

In this paper, the obtained fibers and powder will be termed vascular bundles and parenchyma, respectively. The obtained vascular bundles and parenchyma were checked for mechanical loss by SEM observation. Figure 6 shows the SEM images of the vascular bundle and parenchyma. The vascular bundle consisted of a vessel of approximately 200 μm in diameter and a vascular sheath of approximately 30 μm in diameter as shown in Fig. 6a. The observation of the vascular bundle in the direction parallel to the fiber of the trunk is shown in Fig. 6b, c. There are many small pits near the perforation plate in the vessel and large pits away from the perforation plate. From the appearance of pits, it was thought that inter-vessel pitting is scalariform pitting, the same as those of Japanese bigleaf magnolia (*Magnolia obovata*) (Itoh 1996). Figure 6a–c shows that Zephyr treatment did not cause separation at the cellular level and maintained the tissue structure. The observation of the surface of the vascular bundle is shown in Fig. 6d. Crystalline-like materials were observed on the surface of the vascular bundle. Those were found in the area without parenchyma. Wahab et al. reported that those crystalline-like materials are resin (Wahab et al. 2021a, b). As a result of EDX analysis of the crystalline-like materials, Si was detected as shown in Fig. 6e. As a consequence, crystalline-like materials were found to be silica. Figure 6f shows SEM images of parenchyma. It was confirmed that the parenchyma contained starch averaging about 10 µm in size. Tomimura et al. reported starch content of 55% and 15% in parenchyma and vascular bundles, respectively (Tomimura et al. 1992a). Since the separation method used in this study is a mechanical separation, it was considered possible to separate parenchyma including starch and other substances without chemical degradation. Dried veneer can be mechanically separated into vascular bundles and parenchyma by Zephyr treatment without the use of chemicals. In addition, the length of vascular bundles was shortened by the hammer mill separation used in previous studies because it is a pulverization method (Horiyama et al. 2020). Furthermore, it took time to obtain a large amount of vascular bundles due to the entanglement of vascular bundles shortened by pulverization. Due to the nature of the hammer mill, vascular bundles and parenchyma were obtained together, and separation by a sieve was also necessary.

With the separation by Zephyr treatment used in this study, a large amount of vascular bundles were obtained in a one-time separation if they were dry, and a large amount of vascular bundles and parenchyma were obtained in a short period of time. In addition, long vascular bundles can be obtained without cutting. Furthermore, separation of vascular bundles and parenchyma is easy because parenchyma is sieved off at the bottom of the rollers and only vascular bundles are obtained from the roller exit. Therefore, it is clear that if vascular bundles and parenchyma are mechanically separated, not only can vascular bundles be used with less decay due to the presence of starch, but also the parenchyma, which contains more starch, can be used more effectively.

### Utilization

**Fig. 7:**
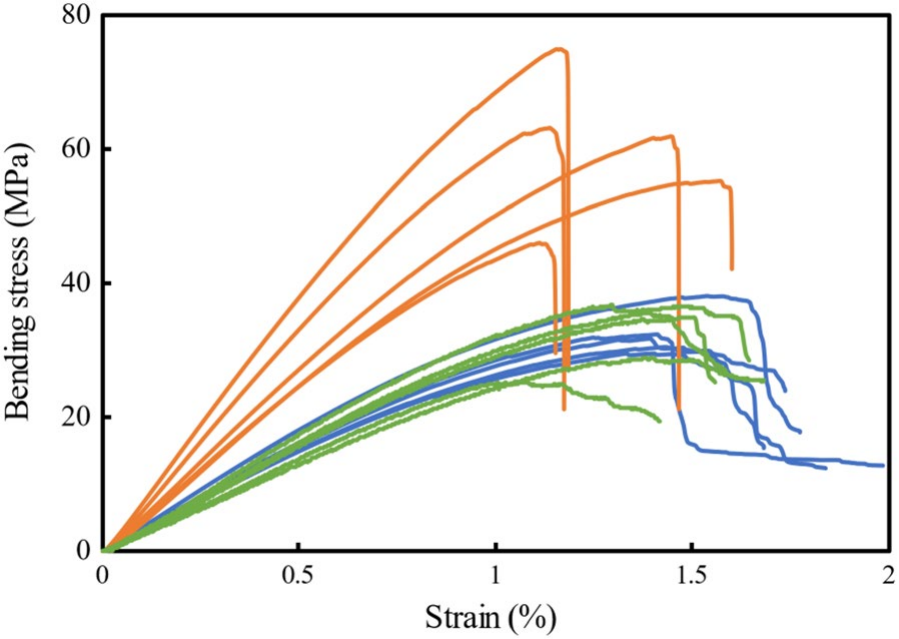
Bending stress–strain curves of particleboards. Orange Line: Long particle, three-layered, Blue Line: Long particle, random, Green Line*: Short particle, random.* The data of previous study (Horiyama et al. 2020)

The vascular bundles obtained in this study were defined as long particles. On the other hand, vascular bundles obtained in a previous report (Horiyama et al. 2020) were defined as short particles. The results of the bending test, internal bond test, and thickness swelling test are shown in Table 2. MOR and MOE of three-layered particleboards with long vascular bundles were 74.2 MPa and 7.3 GPa, respectively. MOR and MOE of three-layered particleboards with long vascular bundles were higher than those of random particleboards with short and long vascular bundles and the JIS criteria values (JIS A 5908: 2015), respectively. MOR and MOE of the OPT were reported to be 5–20 MPa and 0.5–3 GPa, respectively (Srivaro et al. 2018). MOR and MOE of particleboards with the chip of OPT were 30 MPa and 3 GPa, respectively (Hua et al. 2015). MOR of plywood with a veneer of OPT was 30–37 MPa (Mokhtar et al. 2011). MOR and MOE of zephyr strand board were about 20–39 MPa and 3.0–6.0 GPa, respectively (Wardani et al. 2014). Lee et al. reported that particleboard made with only rubber wood particles has better properties than particleboard made with rubber wood and OPT (Lee et al. 2014). The above results indicate that the MOR and MOE of the particleboard with only long vascular bundles prepared in this experiment are much higher than those of previously reported materials made from oil palm. This result indicates that the separation of the oil palm vascular bundles may allow for more expression of the mechanical properties of the vascular bundles. However, based on the results of the random particleboard, the effect of vascular bundle length on MOR and MOE was small. JIS A 5908 (2015) requires that the internal bond (IB) be greater than 0.3% (JIS A 5908, 2015). The results of this study fully satisfied those values. In JIS A 5908(2015), the thickness swelling should be less than 12% (JIS A 5908, 2015). The random particleboards with long vascular bundle resulted in slightly more than 12%. However, the three-layered particleboards with a long vascular bundle were less than 12%, similar to the previous results (Horiyama et al. 2020). Fig. 7 shows the bending stress–strain curves of the particleboards. Three-layered particleboards with long particle were fabricated without significant strain decrease compared to random particleboards with short and long particles. The reason for the existence of variation in the three-layered particleboard was thought to be due to the fact that the orientation of the surface layer was different because it was oriented by hand winding. These results indicate that oriented boards with long vascular bundles can be fabricated with higher-performance boards than conventional boards.

**Table 2:** The average of the density, MOR, MOE, IB, water absorption, and thickness swelling for each particleboard

Particle	Mat	Density (g/cm^3^)	MOR (MPa)	MOE (GPa)	IB (MPa)	Water absorption (%)	Thickness swelling (%)
Long	A three-layered (0° /90° /0°)	0.67 (0.05)	74.2 (12.2)	7.3 (1.0)	1.34 (0.31)	43.74 (2.55)	10.25 (0.79)
	Random	0.70 (0.06)	33.5 (6.5)	3.0 (0.5)	1.42 (0.13)	46.12 (4.14)	12.99 (1.06)
Short	Random	0.71 (0.02)^a^	32.3 (4.8)^a^	3.1 (0.4)^a^	–	43.86 (4.45)^a^	9.73 (0.40)^a^
JIS^b^ 18 type		0.4~0.9	18.0 or over	3.00 or over	0.3 or over	–	12.0 or less

Each value is the average of 5 samples. The value in parenthesis is the standard deviation.

^a^The data of previous study (Horiyama et al. 2020).

^b^JIS A 5908, 2015

There have been several other reports on improving the strength and dimensional stability of OPT by impregnating it with resin or fibering it into MDF (Mangurai et al. 2022; Awang et al 2023). The results of this study suggested that by separating only the long vascular bundles from OPT while maintaining the tissue structure, it is possible to produce boards with low specific gravity and high performance, which was not possible before. In addition, it was observed that the parenchyma obtained by separation contained a large amount of starch. In other words, the separation of OPT into parenchyma and vascular bundles can be effectively utilized by taking advantage of the material characteristics. Pulingam et al. note that OPT left in plantations can be a breeding ground for pests, but that removing OPT from plantations can cause nutrient depletion and increase fertilizer costs (Pulingam et al. 2022). Management of harvested OPT is said to be critical for sustainable production of oil palm. Separation could lead to suggestions for new management methods and utilization of OPT. This will lead to the sustainable use of OPTs waste left in the plantation. Further development of a process to more completely separate the vascular bundles and parenchyma from the OPT is needed. The decay resistance of the resulting vascular bundles is a future study. It had been reported that heat treatment was able to reduce the rate of weight loss due to decay fungi from approximately 13–4.5% (Lee et al. 2018). We plan to compare the results with these heat treatment methods. Further research on effective methods for parenchyma should also be conducted.

## Conclusions

In order to propose a sustainable and advanced utilization of OPTs, the separation of vascular bundles and parenchyma from OPT using Zephyr treatment and the fabrication of boards with the obtained vascular bundles were studied. The results are as follows:

(1) It was found that veneers with low moisture content could be easily separated into vascular bundles and parenchyma by Zephyr treatment. Long vascular bundles were obtained without any breakage that would shorten the length of the vascular bundles. Because of the mechanical separation, parenchyma was also obtained without leaching of starch or other substances. However, it is necessary to examine the separation method to separate the vascular bundles more accurately because parenchyma remains on the surface of the vascular bundles.

(2) The mechanical and physical properties of particleboards made with long vascular bundles performed to JIS A 5908. The MOR and MOE of three-layered particleboards with long vascular bundles obtained by Zephyr treatment were about 74.2 MPa and 7.3 GPa, respectively, which were much greater than those of plywood with palm trunks. Separation of long vascular bundles from OPTs was found to be an effective way to utilize vascular characteristics.

## Data Availability

The datasets generated during and/or analyzed during the current study are available from the corresponding author on reasonable request.

## Abbreviations

OPT: Oil palm trunk
SEM: Scanning electron microscope
MOE: Modulus of elasticity
MOR: Modulus of rapture
IB: Internal bond
TS: Thickness swelling
EDX: Energy dispersive X-ray spectroscopy
